# Determinants of Thyrotoxic Cardiomyopathy Recovery

**DOI:** 10.1155/2013/452709

**Published:** 2013-09-08

**Authors:** Lucia Oliveros-Ruiz, Maite Vallejo, L. Fernando Diez Canseco, Manuel Cárdenas, J. Antonio G. Hermosillo

**Affiliations:** ^1^Departamento de Ecocardiografía, Hospital de Especialidades de la Ciudad de México, Avenida Tláhuac 4866 Del Iztapalapa, 09700 México, DF, Mexico; ^2^Departamento de Investigación Sociomédica, Instituto Nacional de Cardiología “Ignacio Chávez,” Juan Badiano No. 1, Col. Sección XVI, Del. Tlalpan, 14080 México, DF, Mexico; ^3^Departamento de Endocrinología, Hospital Dr. Enrique Cabrera, Avenida Centenario Esquina Prolongación 5 de Mayo, Col ExHacienda de Tarango, Del Alvaro Obregón, 01618 México, DF, Mexico

## Abstract

The purpose was to evaluate the effect of the disease duration prior to treatment, thyroid hormones level, or both on the reversibility of dilated cardiomyopathy. Between January 2006 and December 2010, a longitudinal study with a 6 months follow-up was carried on. One hundred and seventy patients with hyperthyroidism were referred to the cardiologist, and 127 had a 6 months followup after antithyroid treatment and were evaluated by echocardiography. Dilated cardiomyopathy reversibility criteria were established according to echocardiographic parameters. Complete reversibility existed when all parameters were met, partial reversibility when LVEF was ≥55% plus two or three other parameters, and no reversibility when LVEF was ≤55% regardless of other parameters. The results showed that echocardiography parameters related to the regression of myocardial mass were associated with a disease duration shorter than 10.38 months. This was the main predictive variable for reversal of dilated cardiomyopathy, followed by **β**-blocker treatment, and the last predictive variable was the serum level of free triiodothyronine. This study showed that the effect on the myocardium related to thyrotoxicosis was associated with the disease duration before treatment.

## 1. Introduction

Hyperthyroidism's prevalence in Mexico is about 0.7%. Most patients are women between 30 and 50 years old. A hyperdynamic cardiovascular dysfunction is the result of excess thyroid hormone secretion by the thyroid gland, in these patients, manifested mainly by elevated levels of serum triiodothyronine. Thyrotoxicosis involves two sites of action of the thyroid hormone. It acts directly on the heart and on the systemic vasculature producing tachycardia, increased left ventricular mass (LVM), and unchanged systolic function at the beginning of the thyroid dysfunction. However, long-term effects are diastolic dysfunction, cardiac arrhythmias, heart dilation, and a high output heart failure [1, 2].

It is controversial as to whether these cardiovascular alterations due to hyperthyroidism are purely functional or if there is a structural damage. An experimental study in rabbits demonstrated the presence of histological changes in myocytes, and functional damage was more important and related to the hormone levels [3].

In follow-up studies, it has been observed that once hyperthyroidism treatment is started, cardiovascular function returns to normal. However, in a percentage of patients, the cardiac damage remains with poor myocardial function [4, 5].

The purpose of this study is to evaluate the effect of the disease duration, serum thyroid hormones levels, or both on the myocardium of patients with overt hyperthyroidism and the implication of these factors in the reversibility of dilated cardiomyopathy once patients were euthyroid after antithyroid treatment.

## 2. Methods

Between January 2006 and December 2010, a longitudinal 6 months follow-up study was carried on. A total of 2,351 patients were seen in the endocrinology clinic. Of them, 798 were diagnosed with hyperthyroidism in the presence of free triiodothyronine (T3) level >1.59 ng/mL and concomitant suppressed thyroid stimulating hormone (TSH) level <0.35 mUI/mL; [6] 170 of them were referred to the cardiologist with cardiac symptoms, of these, 127 patients were identified with dilated cardiomyopathy and recruited for a 6 months follow-up.

Hypertension, diabetes mellitus, and/or any cardiovascular disease (valvular heart disease, coronary artery disease) were excluded. The disease duration was established in months from the onset of the first symptoms according to the patient until euthyroid state was reached.

The study included four visits to the cardiology clinic: (a) the first visit at referral time, the patients were sent by the endocrinologist because he found cardiovascular alterations, (b) the second visit was when the endocrinologist found the patients euthyroid, (c) and the third and fourth visits were three and six months later, respectively. In each visit a clinical history, physical examination, chest X-ray, electrocardiography (ECG), and echocardiographic assessment were performed, and serum thyroid hormones were determined. 

Echocardiographic assessment was carried out by an experienced operator (L.O.R) using the same echocardiography instrument (Philips Envisor C Philips transducer S3). All digital images were stored on X-Celera system in order to compare images. Left and right ventricular (LVD, RVD) and left and right auricular (LAD, RAD) diameters were measured, and left ventricular ejection fraction (LVEF), left ventricular shortening fraction (LVSF), pulmonary artery systolic pressure, (PASP), left ventricular mass index (LVMI), and left ventricular mass (LVM), left ventricular end-diastolic (LVEDV) and -systolic volumes (LVESV) were determined using standard M-mode echocardiography and Simpson's method according to the American Society of Echocardiography (ASE) recommendations [7].

Dilated cardiomyopathy reversibility criteria for women and men were established according to the following echocardiographic parameter cut points: left ventricular ejection fraction (LVEF) (≤55%), left ventricular diastolic diameter (LVDD) (women ≥47 mm and men ≥49 mm), left ventricular systolic diameter (LVSD) (women ≥31 mm and men ≥35 mm), left ventricular end-diastolic volume (LVDV) (women ≥97 mL and men ≥102 mL), and left ventricular end-systolic volume (LVSD) (women ≥32 (mL) and men ≥34 mL). Complete reversibility was established when all parameters were within established cut points, partial reversibility when LVEF was equal or above 55% plus two or three of the echocardiographic parameters, and no regression when LVEF was less than 55% regardless of other parameters.

Local Research Ethics Committee approved the study protocol, and all participants signed the informed consent form.

### 2.1. Data Analysis

Numerical variables showed a different distribution from normal standards (Gaussian distribution) (test of normality Shapiro-Wilk's, *P* > 0.05). Data are presented as percentiles 25 and 75 and median. Relative frequencies and proportions were estimated for the nominal and categorical variables. Stata 10.0 for Windows software was used for the statistical analysis.

To estimate the effect of disease duration and/or the thyroid hormone levels on myocardium at the referral, regression coefficients were estimated for each echocardiographic parameter (the dependent variables) against disease duration or the level of thyroid hormone (the independent variables). Natural log transformed was used to normalize a skewed distribution. Because a bimodal distribution was identified, LVMI and LVM were divided into low (46–52 and 73–81, resp.) and high values (62–67 and 123–128, resp.). No interaction was identified between the disease duration and the thyroid hormone levels (*P* > 0.20).

The percentage of patients' reversibility status was estimated according to established criteria and compared against disease duration, heart rate, serum thyroid hormones (T3, T4, and TSH), and echocardiographic parameters at referral time (LVDD, LVSD, LVEDV, LVESV, LVEF, LVSF, LVMI, LVM, and PASP). The Kruskal Wallis test was used for the numerical variables and Chi^2^ test or Fisher's exact test as required for the categorical. 

Echocardiographic parameters at referral time and at six months by the reversibility status were compared with the two way ANOVA (natural log-transformed was used to normalize a skewed distribution). The percentage of change of the echocardiographic parameters between referral time and at 6 months was calculated: (the values of the echocardiographic parameters at 6 months minus the parameter at referral time divided by the values of the echocardiographic at referral time) ∗ 100. Statistical significance was set at an alpha level ≤0.05. 

Multivariable analysis was carried out using the classification and regression tree (CART) modeling [8]. This method allows the construction of a binary tree structure classifier that starts with the whole measurement space itself, which by definition is the matrix containing all measurement vectors, and proceeds by repeated splits of subsets of the measurement space into two descendant subsets. The fundamental idea is to select such split in which the data in the descendant subsets are “purer” according to the classification problem. The response variable was the dilated cardiomyopathy recovery classification (complete, partial, or none). The CART was estimated using disease duration, pharmacological treatment, antithyroid treatment, and hormone serum values (T3 and TSH). In order to clarify CART's results, a different geometric figure was used to identify each predictive variable, and the terminal nodes are identified with an asterisk. 

## 3. Results

### 3.1. Findings at Referral Time

Most patients had diffuse toxic goiter (79 patients (62%)), multinodular goiter was found in 32 patients (25%), and only 16 patients (13%) had an autonomously functioning thyroid nodule. Sixty-six (52%) were treated with radioactive Iodine. In 32 patients (25%), the thyroid was removed surgically, and the remaining 29 (23%) received antithyroid drug treatment. Sixty-six patients (52%) were in sinus rhythm, 49 (39%) in atrial fibrillation, and the remaining had flutter or supraventricular tachycardia. Cardiovascular symptoms identified were tachycardia (90%), palpitations (85%), dyspnea (65%), atrial fibrillation (45%), or systolic hypertension (30%). One hundred and two were women (81%), and 25 were men (19%). 

Clinical data, thyroid hormones, and echocardiographic parameters at the first visit at referral time are shown in Table 1. Most patients were young, with a normal to low body mass index (BMI), a long disease duration, and tachycardia. TSH (0.004–0.32 mU/mL) was within abnormal limits, and 83 patients (69%) also had abnormal levels of free T3. Echocardiographic parameters confirmed dilated cardiomyopathy in all patients.

Most altered echocardiographic parameters were associated with disease duration, with a greater effect in those related to decreased myocardial mass (LVEF, LVDV, and LVSV). Free T3 levels were associated with the LVMI and the LVDD (Table 2).

### 3.2. Findings at 6 Months Followup

At the second and third visits, no significant changes were identified. At the 6 months visit, the percentage of patients with complete, partial, or no reversibility of myocardial damage was very similar (31.4%, 31.4%, and 37.2%, resp.); however, analysis by gender showed significant differences; the male group had 50% total recovery, while in the females, complete reversibility was observed in only 26%, and most of them had a partial recovery (40%) (*P* = 0.000). Disease duration since diagnosis was related to reversibility status; those with complete reversibility had less disease duration (13 months) than those with no regression (24 months) (*P* < 0.05). When disease duration was analyzed by gender, there was no difference (Table 3).

**Figure 1:**
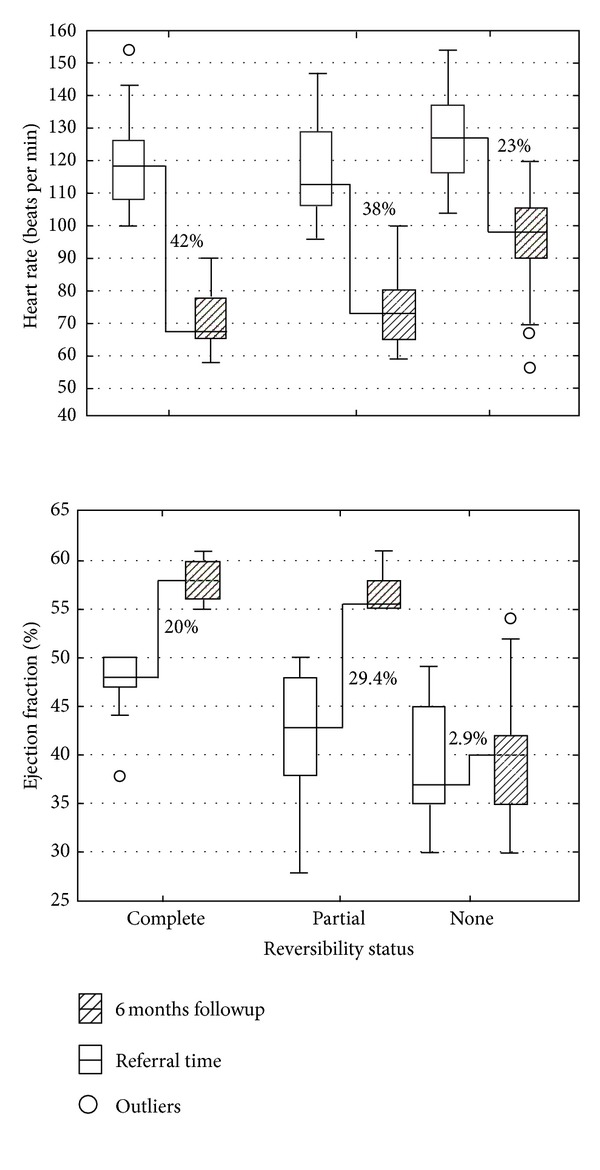
Percentage of change at 6 months followup.

Significant changes were observed only in the LVEF, LVDD, LVSD, LVEDV, and LVESD between the second and 6 months follow-up visits in patients with complete and partial reversibility, but not in those with no reversibility (Table 4). Heart rate and ejection fraction showed significant variations between these two visits. In patients with complete reversibility, heart rate decreased 42% (from 118.5 to 67.5 bpm). A similar situation was observed in patients with partial reversibility (heart rate decreased 38%, from 113 to 73 bpm); in patients with no reversibility, the heart rate only decreased 23% (from 127 to 98 bpm) (upper panel in Figure 1). The ejection fraction returned to normal in all patients with complete reversibility, while in those with partial reversibility, it increased 29.4%, and in patients with no reversibility, the percentage of change was only 2.9% (lower panel in Figure 1). The remaining echocardiographic parameters showed significant changes only in patients with complete and partial reversibility.

Multivariate analysis (CART modeling) showed that disease duration was the main predictive variable in the recovery of dilated cardiomyopathy, followed by pharmacological treatment, and the least important were T3 serum levels.

**Figure 2:**
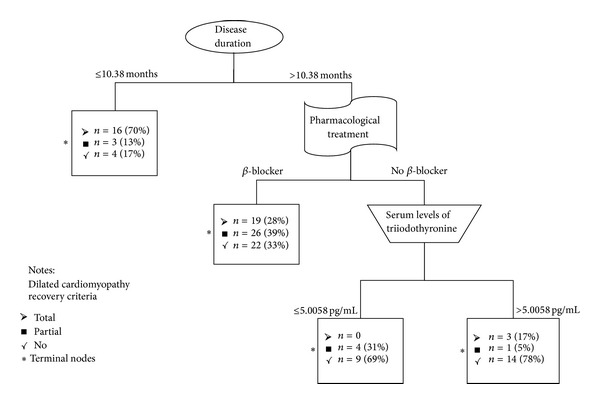
Predictive variables related to reversibility of dilated cardiomyopathy.

Those patients that had a disease duration shorter or equal to 10.38 months recovered only with thyroid treatment within six months (left side of Figure 2); however, when disease duration was longer, they required beta-blocker (*β*-blocker) treatment to achieve complete recovery. Those with a long disease duration (>10.38 months), not receiving *β*-blocker treatment and with elevated levels of serum T3, did not recover or had a partial recovery (right side of Figure 2).

## 4. Discussion

Myocardial damage is directly related to the action of thyroid hormones, increased early in hyperthyroidism producing heart failure, that with time becomes chronic [2, 9–11]. However, most studies agree that once the effect of thyroid hormones is eliminated, myocardial damage may be reversible in patients with less than 6 months disease duration [12–15]. In a recent study, Smit et al. [16] found that even long-term subclinical hyperthyroidism has profound effects on cardiac diastolic function that may disappear when euthyroidism is restored. 

Although the exact mechanism remains unclear, multiple factors are likely to participate in the development of dilated cardiomyopathy and left ventricular systolic dysfunction in patients with hyperthyroidism. Patients with long-standing hyperthyroidism may occasionally have poor cardiac contractility, low cardiac output, and symptoms and signs of congestive heart failure associated with dilated cardiomyopathy (DCM) because of a decreased LVEF, suggesting that the heart undergoes some pathological remodeling [17].

Thyrotoxic heart disease clinical manifestation is characterized by its rapid course and recovery after thyroid dysfunction normalization [1]. In the present study, those patients with longer disease duration had worse myocardial damage represented by left ventricular systolic dysfunction, an increased left ventricular end diastolic size, and a LVEF less than 0.35 and were from the no reversibility group. These results also showed that myocardial recovery was more often observed in men. Most women with dilation of left ventricle persisted with a decreased systolic function 6 months after euthyroid state was achieved despite the treatment used.

Chronic exposure to thyroid hormone may trigger heart failure because of a high cardiac output associated with systolic left ventricular dysfunction with subsequent cardiovascular morbidity by volume overload due to reduced myocardial contractility, which activates sympathetic activity on the heart. 

In this study, the duration of thyroid dysfunction suggests that physiological compensatory and pathological hypertrophy may coexist and lead to a concentric cardiac hypertrophy, in which thyroid hormones may play very different roles from those in eccentric cardiac hypertrophy [13].

Previous studies [12, 15] suggest that if hyperthyroidism is recognized early and treated, dilated cardiomyopathy could be prevented; results in this study showed a negative prognosis once patients had developed dilated cardiomyopathy with impaired left ventricular diastolic function. Therefore, it seems that chronic exposure to thyroid hormones may have more long-lasting hemodynamic changes [18, 19]. 

The no reversibility may not only be related to high levels of thyroid hormones, but could also be the result of an alteration of the sympathetic nervous system with increased catecholamines. This combination could result in a tachycardiomyopathy [20, 21]. 

Antithyroid therapy and *β*-blockers for heart rate control, prevention of heart remodeling, and avoidance of hemodynamic overload will lead to the resolution of heart failure.

## 5. Conclusions

Patient's recovery is mainly dependent on the hyperthyroidism duration previous to treatment. Factors which may play a role in no recovery are no *β*-blocker administration and high T3 serum levels. Therefore, the use of *β*-blocker is an important factor for complete recovery.

## 6. Study Limitations

The real disease duration is unknown; it depends on the patient's recollection at the time of history taking on the first endocrinology visit. Patients included in this study were only those the endocrinologist considered to have a cardiac abnormality. The follow-up was limited to 6 months; therefore, complete reversibility in those with a partial recovery may be achieved later and could not been determined in this study.

## Figures and Tables

**Table 1:** Clinical conditions, thyroid hormones, and echocardiography parameters at referral.

	25th percentile	Median	75th percentile
Clinical variables			
Age (years)	28	33	42
BMI (Kg/m^2^)	22	22.5	23
Disease duration (months)	11	16	25
Heart rate (bpm)	108	120	133
Thyroid hormones			
Free triiodothyronine (T3L) (pg/mL)	3.6	4.8	6.21
Total thyroxine (T4) (ng/dL)	6.8	9.3	12.1
Thyroid stimulating hormone (TSH) (mU/mL)	0.14	0.060	0.08
Echocardiographic parameters			
Left ventricular diastolic diameter (mm)	52	53	56
Left ventricular systolic diameter (mm)	31	32	34
Left ventricular ejection fraction (%)	37	45	48
Shortening fraction (%)	27	29	32
Telediastolic volume (mL)	85	100	105
Telesystolic volume (mL)	33	37	41
Pulmonary pressure (mmHg)	35	43	56
Left ventricular mass index (g/m^2^)	47	49	50
Left ventricular mass (g)	77	78	79

Note: normal thyroid hormones levels: T3L 2.50–3.90 pg/mL, T4T 6.09–12.23 ng/dL, and TSH 0.34–5.60 mU/mL, and normal echocardiography parameters: left ventricular diastolic diameter (LVDD) (women 47 mm and men 49 mm), left ventricular ejection fraction (LVEF) (55%), left ventricular end-diastolic volume (LVDV) (women 97 mL and men 102 mL), left ventricular end-systolic volume (LVSD) (women 32 mL and men 34 mL), pulmonary pressure (30 mmHg), and left ventricular mass (women 110 g/m^2^ and men 134 g/m^2^).

**Table 2:** Association of disease duration or free triiodothyronine (T3) levels on the myocardium.

	*β* coefficient*	95% CI*	*P *value	*R* ^ 2^
Disease duration				
Left ventricular diastolic diameter (mm)	0.056	0.043–0.069	0.000	0.354
Left ventricular systolic diameter (mm)	0.059	0.043–0.073	0.000	0.326
Left ventricular ejection fraction (%)	−0.176	−0.219–(−0.133)	0.000	0.343
Shortening fraction (%)	−0.088	−0.114–(−0.062)	0.000	0.264
Pulmonary pressure (mmHg)	0.271	0.202–0.341	0.000	0.323
Telediastolic volume (mL)	0.124	0.092–0.157	0.000	0.313
Telesystolic volume (mL)	0.103	0.061–0.145	0.000	0.161
Left ventricular mass index (≥62)	−0.022	−0.035–(−0.007)	0.004	0.294
Left ventricular mass index (≤61)	−0.022	−0.033–(−0.011)	0.000	0.121
Heart rate (beats/min)	0.099	0.059–0.140	0.000	0.158
Free T3				
Left ventricular diastolic diameter (mm)	0.023	0.0002–0.046	0.048	0.023
Pulmonary pressure (mmHg)	0.125	0.009–0.240	0.034	0.028
Left ventricular mass index (46–52)	−0.020	−0.035–(−0.005)	0.010	0.056

*Natural logarithmic units.

**Table 3:** Myocardial recovery according to gender and disease duration.

Myocardial reversibility	Gender			*P *value
	Female (*n* (%))		Male (*n* (%))	
Complete	26 (26)		12 (52)	
Partial	38 (39)		0	0.0000*
None	34 (35)		11 (48)	

Disease duration (months)				
	25th percentile	Median	75th percentile	

Complete	8	13	19	
Partial	12	16.5	22	0.000**
None	12	24	28	

*Fishers exact test. **Kruskal Wallis.

**Table 4:** Echocardiographic parameters at enrollment and at the 6 months followup.

Parameters		Referral			6 months	
	25th percentile	Median	75th percentile	25th percentile	Median	75th percentile
Complete reversibility (n = 38)						
LVEF*	45	48	50	55	58	60
LVDD*	50	52	53	43	45	46
LVSD*	30	31	32	27	27	28
LVEDV*	80	84	101	76	80	90
LVESV*	30	32	39	27	30	31
Partial reversibility (n = 34)						
LVEF*	35	43	49	55	56	61
LVDD*	52	54	56	45	47	48
LVSD*	31	32	35	27	27	28
LVEDV*	87	100.5	108	80	94	99
LVESV*	34	40.5	44	30	35	38
No reversibility (n = 49)						
LVEF	33	37	49	32	40	50
LVDD	54	55	56	49	54	57
LVSD	33	34	35	30	33	36
LVEDV	98	103	107	90	110	114
LVESV	35	39	41	30	40	45

*MANOVA ANOVA *P* < 0.05.

Note: LVEF: left ventricular ejection fraction (%), LVDD: left ventricular diastolic diameter (mm), LVSD: left ventricular systolic diameter (mm), LVEDV: left ventricular end-diastolic volume (mL), *LVESV: left ventricular end-systolic volume (mL).
